# Comparison of Estimated Incentives for Preventing Postpartum Depression in Value-Based Payment Models Using the Net Present Value of Care vs Total Cost of Care

**DOI:** 10.1001/jamanetworkopen.2022.9401

**Published:** 2022-04-26

**Authors:** Nathaniel Z. Counts, Margaret R. Kuklinski, Venus Wong, Mark E. Feinberg, Timothy B. Creedon

**Affiliations:** 1 Mental Health America, Alexandria, Virginia; 2 Department of Psychiatry and Behavioral Sciences, Albert Einstein College of Medicine, Bronx, New York; 3 Social Development Research Group, University of Washington School of Social Work, Seattle; 4 Department of General Internal Medicine, Stanford Medicine, Stanford, California; 5 Department of Health and Human Development, The Pennsylvania State University, University Park; 6 Health Equity Research Lab, Cambridge Health Alliance, Cambridge, Massachusetts

## Abstract

**Importance:**

Strong financial incentives are critical to promoting widespread implementation of interventions that prevent postpartum depression. Value-based payment (VBP) approaches could be adapted to capture longer-term value and offer stronger incentives for postpartum depression prevention by sharing the expected future health care savings estimated by reduced postpartum depression incidence with clinicians.

**Objective:**

To evaluate whether sharing 5-year expected savings estimated by reduced postpartum depression incidence offers stronger incentives for prevention than traditional VBP under a variety of circumstances.

**Design, Setting, and Participants:**

This decision analytic model used a simulated cohort of 1000 Medicaid-enrolled pregnant individuals. Health care costs for individuals receiving postpartum depression preventive intervention or not, over 1 or 5 years post partum, in a variety of scenarios, including varying rates of Medicaid churn (ie, transitions to a new Medicaid managed care plan, commercial insurance plan, or loss of coverage) were estimated for the period 2020 to 2025. The model was developed between March 5 and July 30, 2021.

**Exposure:**

Sharing 100% of 1-year actual health care cost saving vs 50% of 5-year estimated health care cost savings associated with reduced postpartum depression incidence.

**Main Outcomes and Measures:**

The main outcome was the amount of clinician incentive shared in a VBP model from providing preventive interventions. The likelihood of the health care payer realizing a positive return on investment if it shared 50% of 5-year expected savings with a clinician up front was also measured.

**Results:**

The simulated cohort was designed to be reflective of the demographics characteristics of pregnant individuals receiving Medicaid; however, no specific demographic features were simulated. Providing preventive interventions for postpartum depression resulted in an estimated 5-year savings of $734.12 (95% credible interval [CrI], $217.21-$1235.67) per person. Without health insurance churn, sharing 50% of 5-year expected savings could offer more than double the financial incentives for clinicians to prevent postpartum depression compared with traditional VBP ($367.06 [95% CrI, $108.61-$617.83] vs $177.74 [95% CrI, $52.66-$296.60], respectively), with a high likelihood of positive return for the health care payer (91%). As health insurance churn increased, clinician incentives from sharing estimated savings decreased (73% reduction with 50% annual churn).

**Conclusions and Relevance:**

In this decision analytic model of VBP approaches to incentivizing postpartum depression prevention, VBP based on 5-year expected savings offered stronger incentives when churn was low. Policy should support health care payers and clinicians to share estimated savings and overcome health insurance churn issues to promote wide-scale implementation of interventions to prevent perinatal mental health conditions.

## Key Points

**Question:**

Do value-based payment (VBP) models that share 50% of the 5-year expected health care savings estimated by reduced postpartum depression incidence offer larger incentives for clinicians to prevent postpartum depression than traditional VBP models that share 100% of 1-year actual savings?

**Findings:**

In this decision analytical model with a simulated cohort of 1000 pregnant individuals enrolled in Medicaid, sharing estimated savings offered more than double the financial incentives for clinicians to prevent postpartum depression than traditional VBP models, assuming continuous health insurance coverage (ie, no churn). This incentive decreased as rates of annual health insurance churn increased.

**Meaning:**

These findings suggest that VBP models that share expected future savings may offer greater incentives for implementing interventions that prevent postpartum depression, but additional policy action is needed to address challenges posed by health insurance churn.

## Teaser

This decision analytical model evaluates whether sharing 5-year expected savings estimated by reduced postpartum depression incidence offers stronger incentives for prevention than traditional value-based payment models under a variety of circumstances.

## Introduction

More than 1 in 8 individuals experience depression during the perinatal period, from pregnancy to 3 months post partum.^1^ These mental health needs are associated with later health concerns, relationship challenges, and lost productivity.^2^ These impacts are also associated with high societal costs, with perinatal behavioral health conditions costing an estimated $14 billion nationally over the first 5 years post partum.^3^ In 2019, the US Preventive Services Task Force (USPSTF) recommended that “clinicians provide or refer pregnant and postpartum persons who are at increased risk of perinatal depression [such as low-income or with subclinical antenatal depressive symptoms] to counseling interventions.”^4,5^ Under the Patient Protection and Affordable Care Act (ACA), USPSTF-recommended preventive services must be covered by most commercial health insurance plans and, under some circumstances, by Medicaid, without cost to individuals—extending access to preventive services for postpartum depression (PPD) for the first time.^6^

Unfortunately, existing payment approaches are not well-suited to promoting the scale up of PPD preventive interventions. Experiences with chronic care management and collaborative care in mental health demonstrate that fee-for-service reimbursement alone may not sufficiently incentivize widespread clinician implementation.^7,8^ Value-based payment (VBP) offers a promising alternative by providing more flexible, quality-based incentives. Most VBP models, including maternity care models and Accountable Care Organizations (ACOs), offer shared savings based on reductions in the total cost of care (TCOC), a measure of how much was actually spent over the episode or past-year compared with a forecasted benchmark based on an individual’s health risk, with payments modified based on clinician performance on quality measures.^9^ To date, VBP models have seen mixed results in reducing overall costs and improving outcomes, which may arise in part because the focus on past-year savings prioritizes short-term cost containment rather than implementation of effective interventions to promote long-term health at scale (such as PPD prevention).^10,11,12^ As most savings from preventing PPD may accrue after the first year,^4^ VBP based on 1-year TCOC may miss much of the opportunity to share value and provide strong financial incentives for implementing interventions to prevent PPD.

One alternative would be to initiate a VBP contract based on the net present value of care (NPVoC).^13^ Building on TCOC, NPVoC forecasts future savings as estimated by improvements in an individual’s health risk over the performance period and shares some of these estimated future savings up front with the clinician. Maryland implemented this model for diabetes prevention, with the Center for Medicare & Medicaid Services (CMS) sharing 5-year estimated health care savings when Maryland achieves reduced diabetes incidence relative to a benchmark.^14^ Reduced incidence of past-year PPD relative to a benchmark would similarly project later health care savings. If payers shared these estimated savings with clinicians, payers may offer substantial and flexible financing for clinician practice transformation toward effective PPD prevention, in addition to the reimbursement received for delivering the specific preventive counseling services. This could make NPVoC-based VBP more effective than TCOC-based VBP for incentivizing implementation of effective PPD preventive interventions.

In this article, we estimate the 5-year health care savings from providing perinatal counseling interventions that prevent PPD in a Medicaid-enrolled population. We then compare an NPVoC-based VBP model that shares 50% of the savings over 5 years with a clinician with a TCOC-based VBP model that shares 100% of the savings over 1 year. We also examine the likelihood that a payer will have a positive return on investment (ROI) after sharing 50% of 5-year expected savings with a clinician up front. We repeat this analysis for different populations, different implementation strategies, and different rates of Medicaid “churn” (ie, transitions to a new Medicaid managed care plan, commercial insurance plan, or loss of coverage).^15^

## Methods

Drawing on the approach in a recent cost-effectiveness study of PPD treatment,^16^ we developed a simulation model to compare potential shared savings payments to clinicians between NPVoC-based VBP and TCOC-based VBP. Our model simulated the development of PPD among 1000 Medicaid-enrolled pregnant individuals without antenatal depression (as individuals with antenatal depression should be identified and receiving treatment)^17,18^ and then simulated their states of being depressed, not depressed, or deceased, along with the associated health care costs to the Medicaid payer, in each month from birth to 5 years post partum. This individual-level discrete time state transition model allowed for intuitive modeling of a simple disease process in a set population.^19^ We followed the Consolidated Health Economic Evaluation Reporting Standards (CHEERS) guidelines in this analysis.^20^ This study analyzed secondary and deidentified data sources and, per the Common Rule, was thus exempted from institutional review board review and the requirement for informed consent.

We used this model to estimate health care savings over 1 and 5 years from 2 prevention implementation strategies: providing preventive counseling (1) universally or (2) to only those who screen positive for subclinical antenatal depressive symptoms. For the first strategy, we also tested 4 additional scenarios: 1a and 1b, with varying 20% and 50% health insurance churn; 1c, including an additional intervention that addresses social needs (eg, financial insecurity or housing instability); and 1d, taking a population perspective that allows for incomplete intervention penetration (ie, not all eligible individuals ultimately receive preventive counseling). For each, we calculated savings by subtracting projected health care costs for a cohort receiving preventive counseling, which decreased the incidence of PPD in the model, from projected health care costs of a cohort not receiving intervention. The eFigure in the Supplement illustrates the decision models.

For each prevention implementation strategy and scenario, we then compared payments to clinicians under 2 VBP approaches: (1) sharing 100% of the savings generated over the first year with clinicians, as in TCOC-based VBP, or (2) sharing 50% of the savings generated over 5 years with clinicians, as in NPVoC-based VBP. For the TCOC-based VBP, 100% shared savings over 1 year was similar to many ACOs, but the 1-year duration was generous: the period for shared savings would often start during pregnancy and only extend several months post partum. For the NPVoC-based VBP, 5 years was similar to the Maryland model for diabetes prevention. Because clinicians do not share the risk of unrealized future savings, we set a conservative shared savings rate of 50%. For the NPVoC-based VBP, we also calculated the likelihood that a payer would realize a positive ROI (eg, whether the payer receives more savings than what was paid out to the clinicians) after sharing 50% of 5-year mean expected savings with a clinician up front.

We calculated intervention costs separately, as they are variable depending on VBP arrangement and context. When a clinician does not provide any intervention at baseline, all interventions count against shared savings unless the VBP arrangement specifically exempts it. As clinicians provide more intervention and the VBP benchmark updates to reflect this, the extent to which an intervention counts against shared savings will reduce.

### Statistical Analysis

The simulation addressed uncertainty in many inputs in the model using a Monte Carlo approach. We ran the simulation 1000 times for each cohort and averaged savings across runs, with model development and analysis conducted between March 5 and July 30, 2021. All analyses were completed in R version 4.04 sing the hesim package.^21^ No statistical significance testing was conducted. Key parameters are summarized below. Parameter estimates and detailed explanations of assumptions are available in eTable 1 and eAppendix 1 in the Supplement, with additional details on model design in eAppendix 2 in the Supplement. Sensitivity analyses are described in eAppendix 3 in the Supplement.

#### PPD Prevalence, Subtype, and Course

We estimated the probability of developing PPD for Medicaid-enrolled individuals without antenatal depression based on the literature.^1,22,23^ We also estimated the probability of developing PPD for those with and without subclinical antenatal depressive symptoms based on studies on screening.^24,25^ A study identified 6 trajectories of depressive symptoms after initial PPD based on initial PPD symptom severity at 2 months post partum (referred to as moderate, marked, and severe) and whether symptoms persist at similar severity at 8 months (episodic or persistent).^26^ We used this information to estimate the probability of individuals having depression in each month after the postpartum period over a 5-year period, allowing individuals to develop or redevelop depression. The model also included the probability of death at delivery and monthly thereafter and reflected that the probability of death is slightly higher for those with depression because of heightened suicide risk.^27,28,29,30^

#### Preventive Intervention

Preventive interventions were provided during pregnancy and lowered the probability of developing PPD. Interventions did not modify the course of PPD once it occurred in the model, and the model allowed for potential fade-out of intervention effects over time. We used the effect sizes of preventive counseling interventions on PPD incidence from a meta-analysis associated with the USPSTF recommendation.^5^ For scenario 1d with incomplete intervention penetration, we estimated a range of probabilities of receiving intervention (0.50-0.60) based on studies in the meta-analysis.

We also explored the combined association of preventive counseling with an intervention to address social needs with PPD incidence (scenario 1c). Interventions to address social needs are challenging to capture in a meta-analysis, in part because they are extremely heterogenous.^31,32^ Instead, we used evidence from a cash transfer policy, assuming some amount of fungibility between social needs interventions and cash (eg, a sufficient cash transfer could address food insecurity in the same way that a social needs intervention would).^33^ We assumed the social needs intervention would be publicly funded and that the costs would not accrue to the health care payer or clinician.

#### Health Care Costs

The model included monthly health care costs for individuals and their children, which were higher when the individual was depressed, based on costs reported in the cost-effectiveness study that provided the conceptual foundation for the present model and other literature specific to the Medicaid population.^16,34,35,36,37,38,39^ While PPD also causes spillovers into increased health care costs for other household members, including partners and other children, we did not include those costs here, nor productivity benefits. We calculated intervention costs when provided as individual counseling (mean [SD], $761.94 [$225.66] per person), group-based counseling (mean [SD], $137.74 [$40.80] per person), and the additional costs of antenatal screening ($2.27) when relevant, based on the Medicaid fee schedule from a representative state and based on a distribution of expected sessions attended, drawn from the meta-analysis of the preventive interventions.^4,40,41^

If individuals disenroll (due to death or churn), health care costs no longer accrue. In scenarios with Medicaid churn (scenarios 1a and 1b), potential annual rates were derived from published literature.^42,43^ All costs were converted to January 2020 US dollars based on the medical Consumer Price Index.^44^ Values are per-person mean savings across 1000 pairs of runs of a simulation model that estimated the per-person costs of PPD over 5 years, with a 3% annual discount rate. Each pair of runs consists of modeling costs when PPD prevention is implemented and when it is not, and savings reflect the difference in expected costs when prevention is implemented. The reported credible intervals (CrIs) reflect the 25th lowest and 25th highest cost differences among the 1000 runs. Negative values reflect pairs of runs in which prevention did not yield savings but rather increased costs relative to no intervention.

## Results

The simulated cohort was designed to be reflective of the demographics characteristics of pregnant individuals receiving Medicaid; however, no specific demographic features were simulated. Shared savings estimates for each VBP approach under different implementation strategies and scenarios (not including intervention costs) are displayed in Table 1, and intervention costs when all spending counts against shared savings are displayed in Table 2. In the first strategy, in which 1000 Medicaid-enrolled pregnant individuals received preventive counseling, NPVoC-based VBP (50% shared savings over 5 years) would pay $367.06 (95% credible interval [CrI], $108.61-$617.83) per individual, more than twice what TCOC-based VBP (100% shared savings over 1 year) would pay on average at $177.74 (95% CrI, $52.66-$296.60). The payer also had a 91% likelihood of seeing a positive ROI even after sharing the 5-year estimated savings with clinicians up front. If all intervention costs counted against shared savings, all VBP arrangements would face likely loss with individual counseling, which would cost $761.94 (95% CrI, $742.24-$770.73) per person. With group-based counseling, which would cost $136.75 (95% CrI, $134.18-$139.33) per person, NPVoC-based VBP would likely offer an incentive and TCoC-based VBP may yield a small surplus.

**Table 1.** Per-Person Discounted Shared Savings From Preventing PPD in Medicaid-Enrolled Individuals Under Different Value-Based Payment Approaches

Prevention strategy or scenario^a^	Savings (95% CrI), $			Positive ROI for payer, %^b^
	Total 5-y savings per person from PPD prevention	Value-based payment approach		
		TCOC: share 100% of 1-y savings	NPVoC: share 50% of 5-y savings	
1, Preventive counseling provided universally	734.12 (217.21 to 1235.67)	177.74 (52.66 to 296.60)	367.06 (108.61 to 617.83)	91
1a, Annual Medicaid churn: 20%	387.58 (−323.86 to 1069.66)	155.58 (46.01 to 269.69)	193.79 (−161.93 to 534.83)	71
1b, Annual Medicaid churn: 50%	197.96 (−407.54 to 853.38)	124.28 (23.98 to 212.02)	98.98 (−203.77 to 426.69)	61
1c, Additional cash payment or equivalent	985.40 (512.80 to 1403.43)	238.18 (122.53 to 335.72)	492.70 (256.40 to 701.72)	98
1d, Imperfect intervention penetration	412.88 (−374.07 to 1140.30)	99.82 (−92.46 to 280.15)	206.44 (−187.03 to 570.15)	64
2, Provided to those with subclinical antenatal depressive symptoms	461.35 (57.91 to 841.92)	110.76 (18.80 to 206.58)	230.68 (28.96 to 420.96)	88

Abbreviations: CrI, credible interval; NPVoC, net present value of care; PPD, postpartum depression; ROI, return on investment; TCOC, total cost of care; VBP, value-based payment.

^a^ Savings were modeled in a Medicaid-enrolled population that did not have antenatal depression and that was provided an evidence-based counseling intervention antenatally to PPD. Scenarios are described in the Methods section. Across all scenarios, intervention costs are excluded and provided separately in Table 2.

^b^ Values represent the percentage of NPVoC-based VBP simulations in which savings remain for the payer after 50% of 5-year savings are shared (paid out) up front with clinician.

**Table 2.** Per-Person Intervention Costs From Preventing Postpartum Depression When All Spending Counts Against Shared Savings in a Value-Based Payment Approach

Prevention strategy or scenario^a^	Cost (95% CI), $	
	Individual counseling	Group-based counseling
1a, 1b, and 1c; Preventive counseling provided universally	761.94 (742.24-770.73)	136.75 (134.18-139.33)
1d, Imperfect intervention penetration	484.38 (452.71-515.89)	87.56 (81.84-93.26)
2, Provided to those with subclinical antenatal depressive symptoms	534.61 (513.01-556.19)	98.50 (94.60-102.41)

^a^ Scenario descriptions appear in the Methods section.

With 20% annual Medicaid churn (scenario 1a), NPVoC-based VBP payments to clinicians were $193.79 (95% CrI, −$161.93 to $534.83), nearly half what was estimated with no churn. Scenario 1b, with 50% annual churn, reduced the incentive by 73% to $98.98 (95% CrI, −$203.77 to $426.69). With 50% annual churn, TCOC-based VBP outperformed NPVoC-based VBP at $124.28 (95% CrI, $23.98-$212.02). Adding a small cash payment to address social needs (scenario 1c) amplified the potential payoff of NPVoC-based VBP by almost 35% to $492.70 (95% CrI, $256.40-$701.72). Lower intervention penetration (scenario 1d) reduced shared savings estimates, although NPVoC-based VBP still offered $206.44 (95% CrI, −$187.03 to $570.15) per person in up-front financing at an intervention penetration rate of 50% to 60%. Intervention costs, when counted against shared savings, would also be lower ($484.38 [95% CrI, $452.71-$515.89] per person for individual counseling and $87.56 [95% CrI, $81.84-$93.26] per person for group-based).

When only Medicaid-enrolled individuals who screened positive for subclinical antenatal depressive symptoms (but not antenatal depression) received preventive counseling (strategy 2), the opportunity for shared savings decreased, with NPVoC-based VBP paying $230.68 (95% CrI, $28.96-$420.96) per individual, 37% less than in the first strategy. Intervention costs that count against shared savings would also be less, with individual counseling costing $534.61 (95% CrI, $513.01-$556.19) per person and group-based costing $98.50 (95% CrI, $94.60-$102.41) per person across a 1000-person cohort. Findings from sensitivity analyses are available in eTable 2 and discussed in eAppendix 4 in the Supplement.

## Discussion

Our simulation found that NPVoC-based VBP may offer a strong ROI for payers and substantially greater incentives for clinicians to implement preventive interventions for PPD than traditional TCOC-based VBP when the rate of churn is low. Under low churn, sharing 50% of 5-year expected savings with clinicians up front may offer PPD prevention incentives that are more than twice those offered by 100% shared savings over 1 year, with an approximately 91% likelihood of positive ROI for payers.

The incentives from NPVoC-based VBP could help support the implementation of effective PPD preventive interventions and finance ongoing innovation and improvement. If a clinician can reach 1000 individuals who experience low health insurance churn with effective PPD preventive interventions, the savings available for clinician incentives may be greater than $500 000. This is important because the costs of implementing the interventions and reaching 1000 individuals are likely to be large. The initial training costs for a PPD prevention program may be more than $50 000 for a large health care system, not including the costs of lost productivity for staff. As the results demonstrated, when intervention costs count against shared savings, clinician incentives diminish dramatically for group-based counseling and clinicians may take a loss with individual counseling. After implementation, clinicians may want to pay for ongoing staff time for outreach or to offer supports for families to facilitate participation. To cover these costs and begin to realize returns from NPVoC-based VBP, clinicians will need initial funding. To avoid disincentives, payers should exempt preventive intervention costs from VBP shared savings calculations or adjust benchmarks to reflect the expected costs. When initial investment remains a barrier, payers could offer up-front financing that is later counted against the shared savings, as with the Medicare ACO Investment Model.^45^ After implementation, the returns will continue to accrue annually to both the clinician and the payer, assuming that effectiveness is maintained, and these funds can be reinvested toward additional services and new approaches to further improve perinatal health.

Our results indicate that clinicians may achieve less shared savings when they limit intervention to those with subclinical antenatal depressive symptoms. However, this is partially offset by lower intervention costs when it counts against shared savings. Our results also reinforce that health insurance churn poses a barrier to the success of NPVoC-based VBP models for financing PPD prevention. In settings with Medicaid expansion and with few payers and little churn, NPVoC-based VBP could be effectively implemented through private contracting between clinicians and payers. Alternatively, when the rate of churn is higher, clinicians could contract with multiple payers using common payment terms. If all payers agree to the same contract terms, then they are likely to achieve average reciprocity even in the face of churn.

Ideally, federal and state policy would address gaps in Medicaid coverage of pregnant and postpartum individuals in nonexpansion states and other churn issues that disproportionately affect low-income perinatal individuals and their children.^46,47,48^ As noted, Maryland has implemented a method similar to NPVoC at a state level, and the Integrated Care for Kids Model from the Centers for Medicare & Medicaid Innovation (CMMI) is well positioned to implement NPVoC-based VBP for PPD prevention.^49^ To facilitate uptake, CMMI could build in options for NPVoC-based incentives for PPD prevention into its models, provide technical assistance to states, and work with insurance companies to align incentives and overcome churn. The federal government could also support the development of a new generation of cost measures that determine shared savings algorithmically for NPVoC-based VBP using claims data and quality measure reporting, standardizing NPVoC methodologies to make contracting more straightforward across payers.^50^ In all of these efforts, it will be important to risk adjust benchmarks appropriately so that clinicians are incentivized to address those individuals that experience the greatest need.

Finally, our results indicate that combining preventive counseling interventions with interventions that effectively address social needs—cash transfers or their equivalent—can lead to even greater gains under NPVoC-based VBP. As health care increasingly focuses on addressing social needs, NPVoC-based VBP could provide additional flexible incentives for transformation.^51^

Future studies should consider longer time frames for sharing expected savings; sharing financial benefits outside of health care, such as productivity benefits; and begin to include nonmonetizable benefits to individuals as part of the incentives. While this article sought to model the current health care financing environment, value beyond 5 years, value that accrues after health insurance churn, and other sources of value are not captured when benefits are narrowly confined to what can be monetized in health care. A model that takes a broader societal perspective could promote more effective long-term policy making, rather than focusing on what might be feasible in VBP today.

Future studies should also examine the equity dimensions of VBP models for preventing PPD. Future research should analyze how antiracist policies and practices could be incentivized through NPVoC-based VBP models.^52^

Finally, future studies should expand beyond PPD. PPD is only a single condition within perinatal behavioral health, and only one of many needs that must be addressed to improve well-being and achieve life-course health equity. Many of the same ideas examined in this paper are equally applicable to other conditions, and future research should explore these opportunities.

### Limitations

This study has limitations. Our model drew from available literature to simulate various scenarios in a hypothetical cohort using a series of assumptions and may not be applicable to a particular group of individuals receiving specific PPD prevention interventions. The limitations posed by the assumptions made in constructing the model are discussed in greater detail in eAppendix 1 in the Supplement. The discussion of the sensitivity analyses in eAppendix 4 in the Supplement urges caution in relying on the model estimates in settings with high churn rates. Additional research is needed to examine the 5-year cost dynamics associated with specific PPD preventive interventions delivered at scale in diverse Medicaid populations in the US.

## Conclusions

Innovative approaches to financing will be needed to incentivize the implementation of effective interventions to prevent perinatal mental health conditions at scale. The findings of this study suggest that NPVoC-based VBP offers the opportunity to invest substantial resources in clinicians to effectively prevent perinatal mental health conditions while achieving savings for health care payers. Federal and state policy action could accelerate NPVoC-based VBP uptake by supporting implementation and helping to overcome issues related to health insurance churn.

## Notes

Supplement. **eFigure.** Decision Model for Scenarios and Strategies Simulated **eTable 1.** Parameters Used in the Postpartum Depression (PPD) Prevention Simulation Model and Citations to Explanation of Assumptions in eAppendix 1 **eAppendix 1.** Key Parameters and Model Assumptions **eAppendix 2.** Additional Details on the Model Design **eAppendix 3.** Design of Sensitivity Analyses **eTable 2.** Findings from Sensitivity Analyses **eAppendix 4.** Discussion of Sensitivity Analyses
eReferences.
