# Supplementary material for: Comparison of Estimated Incentives for Preventing Postpartum Depression in Value-Based Payment Models Using the Net Present Value of Care vs Total Cost of Care
Source: JAMA Netw Open. 2022 Apr 26;5(4):e229401. doi: 10.1001/jamanetworkopen.2022.9401 (PMC9044113; doi:10.1001/jamanetworkopen.2022.9401)
Supplement: Supplement. — eFigure. Decision Model for Scenarios and Strategies Simulated eTable 1. Parameters Used in the Postpartum Depression (PPD) Prevention Simulation Model and Citations to Explanation of Assumptions in eAppendix 1 eAppendix 1. Key Parameters and Model Assumptions eAppendix 2. Additional Details on the Model Design eAppendix 3. Design of Sensitivity Analyses eTable 2. Findings from Sensitivity Analyses eAppendix 4. Discussion of Sensitivity Analyses eReferences. [file jamanetwopen-e229401-s001.pdf]

Supplemental Online Content

Counts NZ, Kuklinski MR, Wong V, Feinberg ME, Creedon TB. Comparison of estimated incentives for preventing postpartum depression in value-based payment models using the net present value of care vs total cost of care. *JAMA Netw Open.* 2022;5(4):e229401. doi:10.1001/jamanetworkopen.2022.9401

eFigure. Decision Model for Scenarios and Strategies Simulated

eTable 1. Parameters Used in the Postpartum Depression (PPD) Prevention Simulation Model and Citations to Explanation of Assumptions in eAppendix 1

eAppendix 1. Key Parameters and Model Assumptions

eAppendix 2. Additional Details on the Model Design

eAppendix 3. Design of Sensitivity Analyses

eTable 2. Findings from Sensitivity Analyses

eAppendix 4. Discussion of Sensitivity Analyses

eReferences.

This supplemental material has been provided by the authors to give readers additional information about their work.

© 2022 Counts NZ et al. *JAMA Network Open.*

eFigure. Decision Model for Scenarios and Strategies Simulated

Decision Tree for Strategy 1 in which all individuals receive in intervention

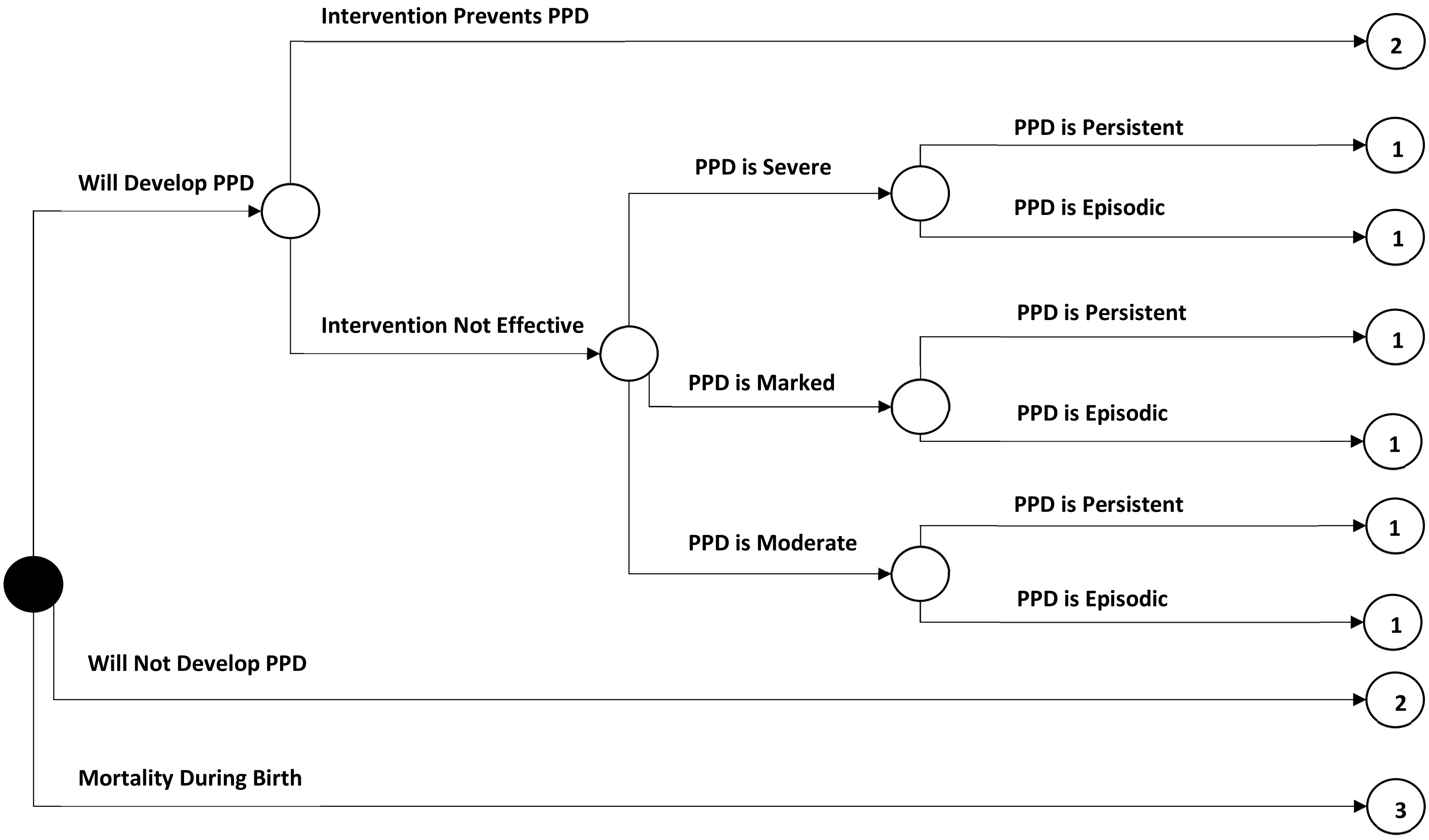

© 2022 Counts NZ et al. *JAMA Network Open.*

Decision tree for Strategy 1, Scenario C, in which there in incomplete intervention penetration

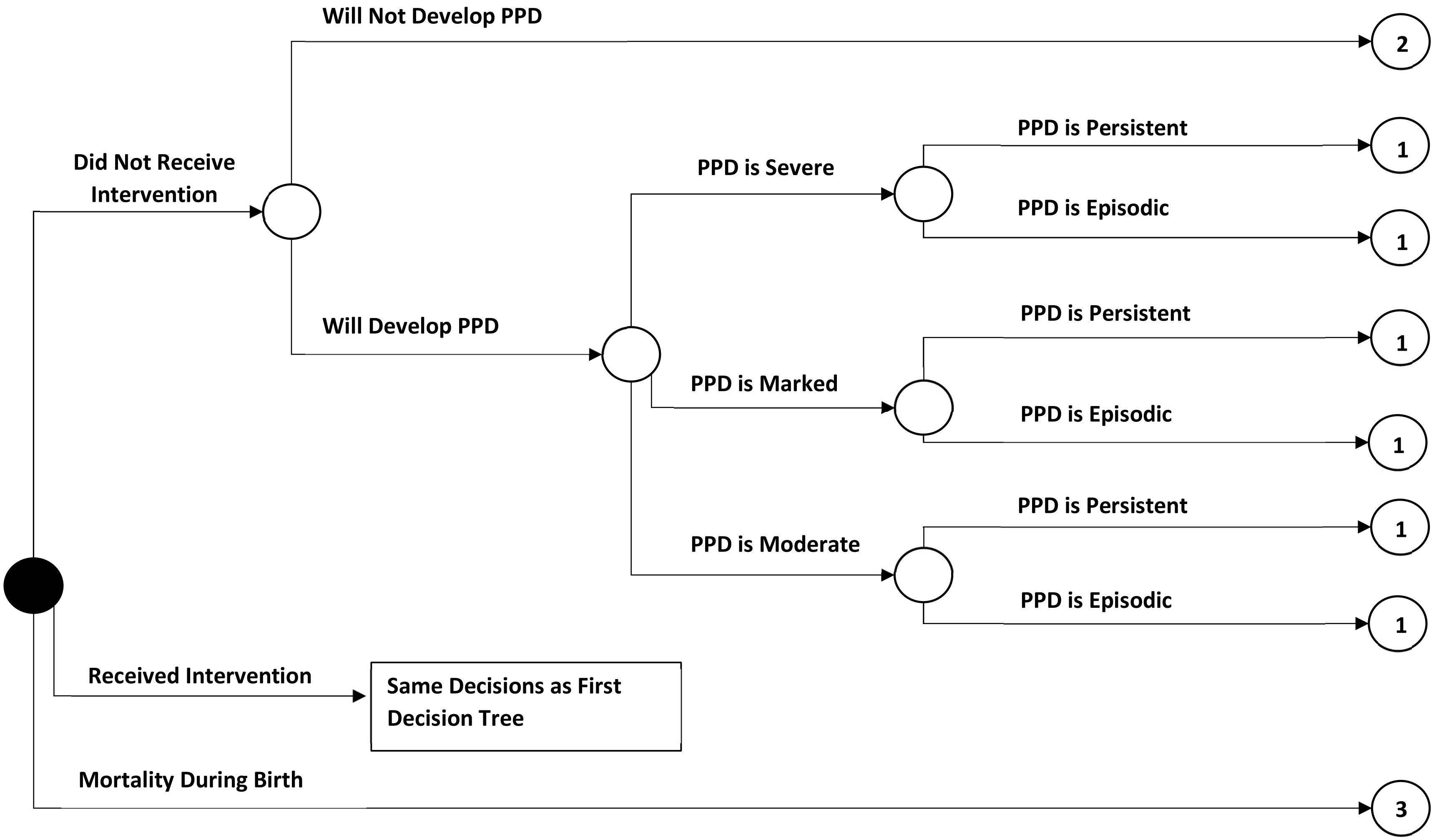

© 2022 Counts NZ et al. *JAMA Network Open.*

Decision tree for Strategy 2, in which all individuals that screen positive for antenatal depressive symptoms receive intervention

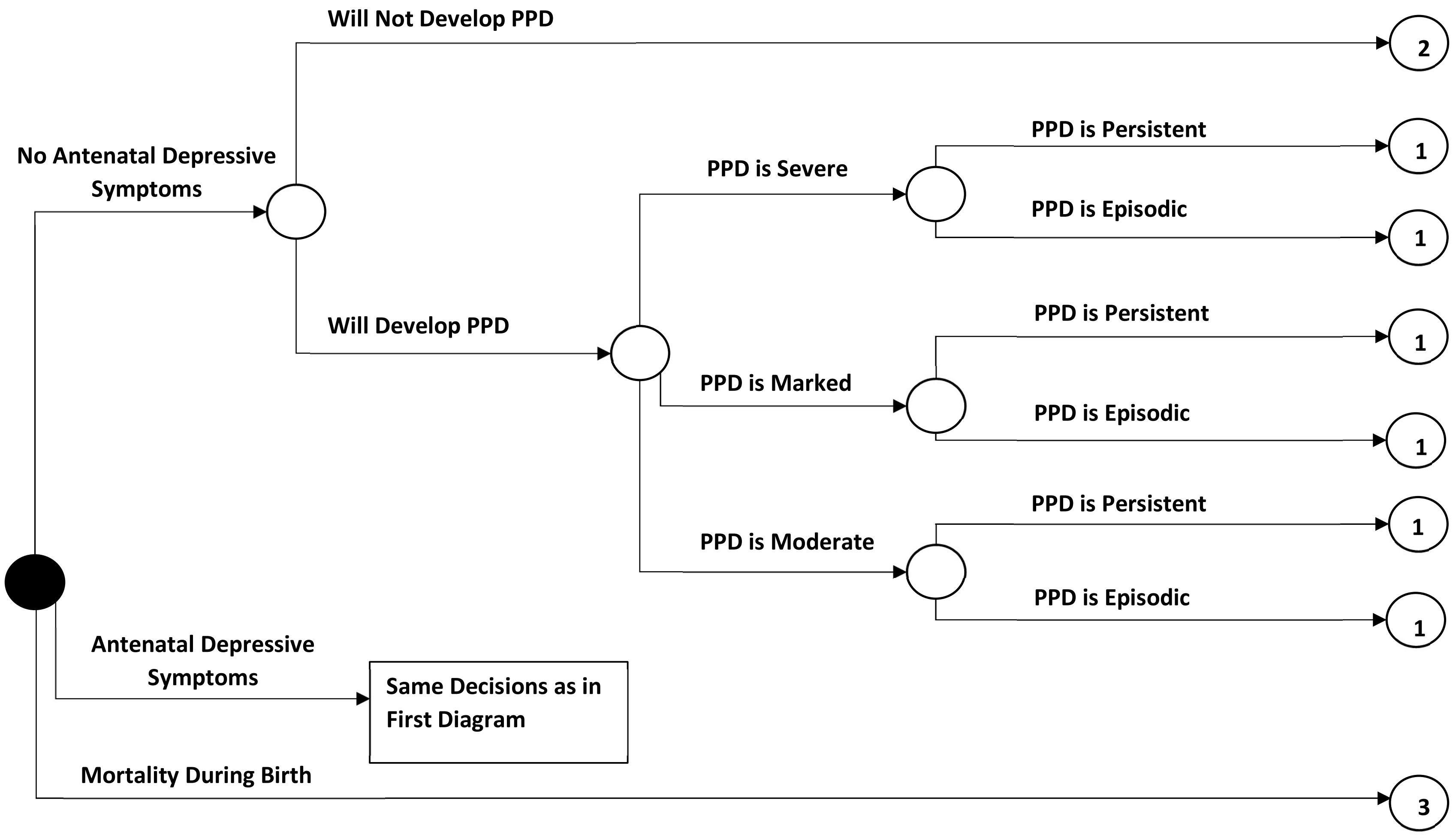

© 2022 Counts NZ et al. *JAMA Network Open.*

Markov Chain

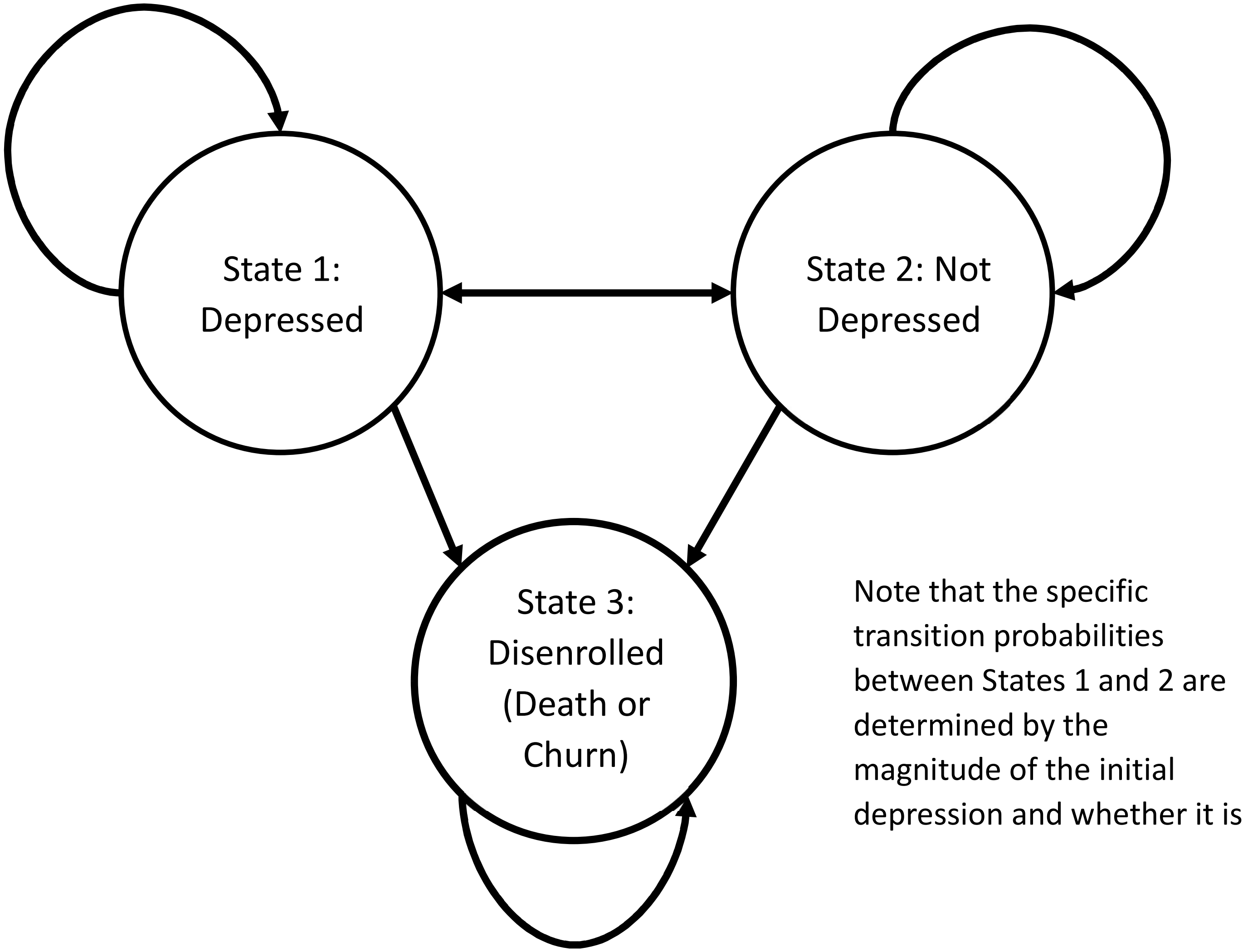

© 2022 Counts NZ et al. *JAMA Network Open.*

eTable 1. Parameters Used in the Postpartum Depression (PPD) Prevention Simulation Model and Citations to Explanations of Assumptions in eAppendix 1

**Probability of Developing PPD**	
**Intervention Strategy or Scenario**	**Probability Distribution**
PPD among those without antenatal depression, Medicaid-enrolled	0.16–0.18 (U) [1]
PPD in the antenatal depressive symptoms screening strategy	0.08–0.09 (U) [2]
PPD among those with subclinical antenatal depressive symptoms	0.16–0.18 (U) [2]
PPD among those without subclinical symptoms	0.04–0.05 (U) [2]
Screening positive for subclinical antenatal depressive symptoms	0.60–0.66 (U) [2]

**Probability for Developing PPD Subtypes among those with PPD** [3]			
Marked & Episodic	0.32 (Remainder) [3]	Marked & Persistent	0.13 (0.01) (B) [3]
Moderate & Episodic	0.16 (0.01) (B) [3]	Moderate & Persistent	0.08 (0.01) (B) [3]
Severe & Episodic	0.23 (0.01) (B) [3]	Severe & Persistent	0.08 (0.01) (B) [3]

**Probability of Depression Each Month** (Interpolated Between these Points)* [4]					
Month	2	8	21	33	60
No Initial PPD	0.007 (B)	0.003	0.021	0.040	0.042
Marked & Episodic	0.804	0.050	0.145	0.238	0.239
Moderate & Episodic	1.000	0.119	0.347	0.305	0.292
Severe & Episodic	0.996	0.260	0.365	0.356	0.340
Marked & Persistent	0.841	0.790	0.462	0.544	0.521
Moderate & Persistent	1.000	0.937	0.656	0.503	0.472
Severe & Persistent	0.994	0.995	0.746	0.645	0.631

**Death Probabilities Each Month** (Probabilities Between Months Interpolated) [5]			
Timepoint	Birth	Month 1	Month 60
Mortality probabilities	2.61x10^{-4}	6.49x10^{-5}	6.94x10^{-5}
Suicide when depressed	1.7x10^{-6}–3.3x10^{-6} (U)		

**Intervention Effects on PPD Probabilities** (Relative Risks)	
Counseling interventions for PPD prevention	0.61 (95% CI 0.47, 0.78) (LN) [6]
Accessing the PPD preventive intervention	0.50–0.60 (U) [6]
Addressing Social Needs	0.77-0.78 (U) [6]

**Intervention Costs per Person**	
Individual Counseling	$761.94 (225.66) (U) [7]
Group-Based Counseling	$137.74 (40.80) (U) [7]
Antenatal Depressive Symptoms Screening	$2.27 [7]

**Healthcare Costs per Month per Person** (in 2020 dollars) [7]			
Health State	Person	Month	Costs
Not Depressed	Mother	<12	$242.36 (36.06) (G)
		≥12	$420.14 (5.28) (G)
	Child	All	$64.45 (0.01) (G)
Depressed	Mother	<12	$624.82 (269.05) (G)
		≥12	$993.03 (9.33) (G)
	Child	All	$77.35 (8.21) (G)
Discount Rate	3% Annually		

U=Uniform Distribution, N=Normal Distribution, G=Gamma Distribution, B=Beta Distribution. Remainder = This probability was calculated by subtracting the other probabilities from one.

* Standard errors too small to be displayed, see eAppendix 1 for methods. The probabilities displayed are before the probability of death is subtracted. Month one probability is extrapolated (maximum of one).

Note: Citations in the table refer to corresponding sections of eAppendix 1, which furthers explains how the parameters were determined and relevant assumptions.

© 2022 Counts NZ et al. *JAMA Network Open.*

eAppendix 1. Explanation of Assumptions for Key Parameters

This section provides additional details on how parameters in the model were determined, the assumptions that the parameters rely on, and their limitations. The numbered sections correspond to references in eTable 1, which provides the parameter estimates and ranges.

General Note on Identification of Studies for Extracting Parameters

Parameters were identified through searches of PubMed and Google Scholar to identify the most recent study that provided representative data on the population of interest and that appeared to be of sufficient methodological rigor. Meta-analyses were preferred over representative single studies. Where only non-representative, single studies were available, we tried to identify at least two studies to incorporate into our parameter estimates and allowed wider variation in the parameter, encompassing the results from both studies, in the Monte Carlo simulation.

1. Probability of Developing PPD for those without Antenatal Depression

A recent meta-analysis estimated that the prevalence of PPD in the US is 0.13 (95% CI: 0.13, 0.14).[1] However, the studies in the meta-analysis used differing cut-points on the Edinburgh Postnatal Depression Scale (EPDS), the primary screening instrument for PPD we rely on in this study. The average EPDS cut-point in the studies meta-analyzed was 11.2, rather than the cut-point we apply of 13. This may cause us to slightly overestimate the prevalence of PPD in the general population. We thus use a uniform distribution of 0.11 to 0.13, which incorporates a lower bound that is approximately in line with how the meta-analysis finds that differing cut-points impact the prevalence estimate. There is also extremely high geographic variability in PPD prevalence in the US population, so this estimate should be viewed as illustrative.[2] We find that the prevalence of PPD in a Medicaid-enrolled population is approximately double the general population.[3] Thus, we double the probability of developing PPD, given that our focus on Medicaid.

One study finds that 26% of women with antenatal depression (using a EPDS cut-point of 12) go on to experience PPD while 6% of those without antenatal depression experience PPD,[4] while another study found similar values for antenatal depression in late pregnancy and later PPD, but with extremely high loss to follow-up.[5] We assume a similar rate of antenatal depression prevalence in our sample as in the studies. Using these estimates, we ensure that the combined PPD prevalence across those with and without antenatal depression is line with our total estimated prevalence above. This offers us a distribution of 0.16 to 0.18 for the probability of developing PPD in the population without antenatal depression in Medicaid. Note that neither sample was from a US population, and may not be representative of depression dynamics in the US context.

© 2022 Counts NZ et al. *JAMA Network Open.*

2. Probability of Developing PPD for those with Subclinical Antenatal Depressive Symptoms

One recent study on antenatal depressive symptoms and later risk for PPD found a positive predictive value of 10.7 and negative predictive value of 97.0 for later PPD for scoring at ≥5 antenatally on the EPDS, but used a cut-point of 10 rather than 13 for depression and removed all women with an antenatal score over 10 from the sample.[6] Another found a positive predictive value of 15.9 and negative predictive value of 96.0, but also used a cut-point of 10 for PPD on EPDS and did not remove women with antenatal depression from the sample.[7] Both also found lower prevalence of PPD in their sample than our estimate, even given the lower cut-point. Guided by these estimates and adjusting to ensure that the final PPD prevalence aligns with our assumed prevalence in the sample of individuals without antenatal depression, we assume that women who screen positive for subclinical depressive symptoms will have a probability of 0.16 to 0.18 (uniformly distributed) of developing PPD, and those who screen negative have a probability of 0.04 to 0.05 of developing PPD. To ensure these probabilities result our expected PPD prevalence, we estimate that individuals have a 0.30 to 0.33 probability of screening positive for subclinical antenatal depressive symptoms.

3. Probability of Developing a Particular PPD Subtype

We assign probabilities of women with PPD developing one of six subtypes ("marked," "moderate," or "severe" based on symptom severity at two months postpartum, as well as "persistent" or "episodic" based on whether symptoms fall within the same severity range at eight months postpartum) based on the proportions given in the study of the course of depressive symptoms over time.[8] We allow each to vary with a beta distribution based on the sample sizes given in the study, except for "marked depression but not persistent," which we set to one minus the probability of the other subtypes. This sample was not from a US population, so caution must be exercised when applying these estimates in the US context.

4. Health State Transition Probabilities in the Model

The same study that gave the PPD subtype proportions also give the EPDS symptom distributions at two months, eight months, thirty-three months and eleven years for each subtype:

Months	2	8	21	33	132
No PPD	4.71 (3.39)	4.14 (3.27)	4.78 (4.03)	5.36 (4.37)	5.03 (4.80)
Moderate & Episodic	13.42 (0.49)	7.64 (3.25)	8.17 (4.56)	9.63 (4.72)	9.09 (5.53)
Marked & Episodic	15.44 (0.50)	8.97 (3.42)	10.75 (5.70)	10.48 (4.93)	9.09 (6.03)
Severe & Episodic	19.06 (2.30)	10.26 (4.25)	11.07 (5.60)	10.87 (5.77)	9.93 (5.84)
Moderate & Persistent	13.50 (0.50)	15.39 (2.96)	12.52 (5.00)	13.55 (5.00)	12.45 (5.64)
Marked & Persistent	15.45 (0.50)	17.8 (3.14)	15.05 (5.09)	13.04 (5.34)	11.52 (5.32)
Severe & Persistent	20.66 (3.05)	19.95 (2.67)	16.29 (4.98)	15.30 (6.19)	14.49 (6.13)

We use the mean and standard deviation of the scores at each time point, assuming a normal distribution of scores, to calculate the probabilities of being above and below a score of 13 at each timepoint. In real life sample, the scores are certainly not normally distributed, but we do not have further information about the distribution. We then fit a series of linear regressions to each pair of points to interpolate the probability of being depressed or not at each month through sixty months, and extrapolate probabilities at month one (capping them at 1). We assume that each of these probabilities follows a beta distribution and we vary the probabilities at these timepoints, based on the sample size in each of the PPD subtypes, for each interpolation/extrapolation.

5. Disenrollment Transition Probabilities in the Model, Including Churn

For baseline mortality, we assume an average age entering the model of 29 (the average age of individuals giving birth in 2019) and use the Social Security Administration's Actuarial Life Tables from 2017, which give the probability of death for each age and gender.[9,10] We convert the annual probabilities at ages 29 and 33 into monthly probabilities and linearly interpolate to give probabilities of natural mortality each month. We also allow for an increased likelihood of death in the perinatal period based on the national maternal mortality rate estimated for 2018,[11] which we increase by 50% in the Medicaid population based on a study on relative risk for maternal mortality by area socioeconomic deprivation in 2018.[12]

We also want to represent the slightly increased risk of death from suicide that results from postpartum depression. Studies have found that the rate of death by suicide among postpartum women may be approximately 2 to 4 per 100,000,[13,14] and another study estimated that approximately half of suicides are completed by individuals with depression.[15] We use this to estimate that the rate of women with postpartum depression dying by suicide is approximately 1 to 2 per 100,000, which we convert to a monthly probability that is applied to the depressed state throughout (although this may overestimate the likelihood of suicide at later timepoints).[16] To represent a Medicaid-enrolled population, we double this likelihood based on findings from statewide analysis in California.[17]

For health insurance churn likelihoods, we use a 20% and 50% annual probability, and convert to monthly probabilities, guided by estimates from the literature.[18,19] Individuals experience churn for a number of reasons. They may opt to (or be forced by state procurement processes to) switch Medicaid managed care plans, they may enroll in employer-sponsored insurance, purchase health insurance on the individual market place (depending on their income and eligibility for premium assistance), or unfortunately they may lose insurance entirely. Pregnant individuals face unique challenges related to Medicaid churn. Prior to the Affordable Care Act (ACA) passage, many postpartum individuals lost coverage when their eligibility due to pregnancy ended at sixty days postpartum. With the ACA, many states expanded Medicaid, providing an additional basis of eligibility for pregnant individuals, which has decreased the rate of churn for pregnant individuals on Medicaid in these states.[20] In states that did not expand Medicaid, postpartum

© 2022 Counts NZ et al. *JAMA Network Open.*

individuals frequently lose coverage as they have fewer options for eligibility and, across all states, churn continues to remain an issue because the generosity of the eligibility category for pregnancy may be higher than other options for coverage.[21] The American Rescue Plan Act of 2021 gave states the option to extend pregnancy-related eligibility to one year postpartum, which offers additional critical support to postpartum individuals, but would still result in churn that undermines the five-year savings window analyzed here, if they do not have another basis for Medicaid eligibility. Churn in general continues to pose a key health equity issue, as it disproportionately impacts low-income individuals, for whom fluctuations in income or employment affect program eligibility, and has disproportionate impacts on postpartum individuals and children, who experience documented gaps in access to care needed for healthy life-course development.[22,23,24]

6. Effects of Interventions on Probability of Developing PPD

To estimate the effect of counseling interventions on probability of developing PPD, we use a meta-analysis that offers the relative risk of developing PPD across included studies for groups that received preventive counseling.[25] The counseling interventions included in the meta-analysis are diverse, but generally involve a series of weekly sessions of cognitive-behavioral therapy or interpersonal therapy delivered by a trained practitioner.[26] There is reason for caution in using these estimates in our simulation. Studies used different EPDS cut-points for depression, many focused on specific subpopulations (e.g. only very low-income individuals or only immigrants from a specific Central American country), were only applied to indicated populations using different screening approaches than we use in our model, and some studies were small. The meta-analysis does take into account the high rate of dropout among individuals that initiate a treatment. Other economic models discount these literature derived estimates to reflect this need for caution.[27] We use the estimate as illustrative, but these may or may not be replicable in different real-world settings.

For the social needs intervention, we rely on a recent systematic review of cash transfer policies in high income countries.[28] One study in the US specifically examined the impact of cash transfer programs on maternal depression among women with children with no more than a high school education at a national scale and found significant effects.[29] The study found that the Earned Income Tax Credit (EITC) reforms in 1990, which is linked to work and restricted to low-income individuals, reduced depression in mothers by 14% of a standard deviation. Most of this effect was concentrated in married mothers who saw a decrease of 20% of a standard deviation, likely in part due to the EITC being contingent on working, which subjected unmarried mothers to additional stress (EITC was associated with substantial increases in labor force participation among unmarried mothers but little increase among married mothers). The size of the 1990 EITC expansion is variable, but the maximum increased from $851 to $1,235 for a family with two or more children in the most generous bracket, and increased in subsequent years.

© 2022 Counts NZ et al. *JAMA Network Open.*

There is likely substantial overlap between the Medicaid and EITC population from the study,[30] although approximately 40% of women in the Medicaid-enrolled population have more than a high school education and these women would have been excluded from the EITC analysis.[31,32] To illustrate a range of relatively credible impacts of a social needs intervention on the same scale of the 1990 EITC reform (but not tied to work), we use a uniform distribution between 14% and 20% of a standard deviation of impact.

To apply this in the context of our model, we calculate the distribution of EPDS scores among individuals who develop PPD in our model (see below for distributions), based on a simulation of 10,000 individuals. This yields a mean EPDS score of 15.88 with a standard deviation of 3.01. If these simulated scores are uniformly reduced by 14% and 20% of a standard deviation, we obtain relative risks of 0.79 and 0.78 respectively. We assumed that this impact was directly additive with the preventive counseling intervention. There is evidence to support that these interventions would be at least partially additive, although the precise interaction effects of different interventions require further study.[33]

7. Healthcare Costs in the Model

We estimate intervention costs using published literature and the Medicaid fee schedule from a representative state. Counseling interventions included in the meta-analysis were delivered individually, in a group, or in a combination.[26] The median number of sessions was 8 with an interquartile range (IQR) of 5 to 11 and the median hours of contact was 12 with a IQR of 4 to 23.3 hours. For simplicity in modeling potential intervention costs, we assumed that clinicians either delivered eight sessions of 60-minute individual therapy (CPT code 90837) or delivered group therapy (CPT code 90853). The meta-analysis also noted "that generally between half to three-quarters of all possible sessions were attended, across all participants, although adherence information was not always available, and reporting was quite variable."[26] Based on this, we created a parameter for the number of sessions attended as a normal distribution with the mean sessions attended at 5 with a standard deviation of 1.5, with limits of one or eight sessions attended. When administering the screen for antenatal depressive symptoms, this can sometimes be included in the costs of the well-visit or can be billed separately, often as CPT code 96160.[34] We assumed it was billed separately to illustrate the potential costs. For the price of each service, we identified Virginia as having a Medicaid fee schedule that is close to, but slightly above, the national average for Medicaid fees, and used Virginia's fees to illustrate potential costs in our model.[35,36] In Virginia, individual counseling is reimbursed for $151.51, group-based counseling is reimbursed for $27.39 per person in the group, and screening is reimbursed for $2.27. We used the distribution of attended sessions and the price per session of each delivery type to estimate the distribution of per person costs for the entire cohort under each prevention strategy and scenario.

For other healthcare costs in the model, we relied on cost estimates from published studies. The cost-effectiveness study estimated costs based on the incremental costs of PPD in the first year postpartum,[37] costs associated with adult depression in general,[38] and the costs related to PPD

© 2022 Counts NZ et al. *JAMA Network Open.*

for children.[39,40] Although some of these studies were based on older data, more recent estimates were quite similar.[41] We then sought to adjust costs to reflect spending in a Medicaid-enrolled population, but found that incremental depression costs are similar between Medicaid and commercial populations,[42] and between the Medicaid population and those used in the cost-effectiveness study that served as the basis for our estimates.[43]

eAppendix 2. Additional Details on the Model Design

All analyses were completed in R version 4.04 using the *hesim* (Health Economic Simulation Modeling and Decision Analysis) package.[44] Note that we represented the cohort of individuals as 1000 different individual samples in the model rather than 1000 patients in the same sample, so randomization for the Markov Chain Monte Carlo process underlying the simulation took place at the level of the individual. We also simulated individuals taking on discrete values generated by the probabilities at each time point. This allows us to express our results as a range of real values determined by the 1000 runs for the 1000 simulated women. Because we have a short cycle length in the context of healthcare utilization, we do not implement a half-cycle correction.[45]

For our results, we calculated the difference in total discounted costs over one or five years between a pairing of a cohort of 1000 individuals that received preventive intervention and a cohort that did not receive intervention, with 1000 such pairings generated through runs of the model. We then calculated the mean difference in costs between the paired cohorts across the 1000 runs, and determined the 95% credible interval by selecting the 25th largest and the 975th largest runs.

© 2022 Counts NZ et al. *JAMA Network Open.*

eAppendix 3. Design of Sensitivity Analyses

We conducted two sensitivity analyses to test the robustness of two central model parameters: the effectiveness of the intervention and the costliness of spending attributable to depression.

One assumption in the model is that the counseling interventions will produce similar effects when implemented in the real world as they did in the meta-analysis. As noted, the studies included in the meta-analysis may not be representative of the general Medicaid population, and interventions are often not implemented with the same fidelity in the real world as in research settings. To test the robustness of the model to overestimation of intervention effects, we run a sensitivity analysis in which counseling interventions only decrease the risk of developing postpartum depression (PPD) by half as much as they did in the meta-analysis.

Another assumption is that the individuals experiencing depression in our cohort are as costly as in the reference studies, which were from a similar demographic for the first year of costs postpartum, but then were taken from the general population. If perinatal individuals incur fewer costs as a result of depression through the first five years of life than we assumed, then we may overestimate cost savings associated with prevention. To test the robustness of the model to overestimation of cost impacts, we run a sensitivity analysis in which the costs that the individual accrues when they are in a depressed state are half as large when compared to costs in the non-depressed state.

© 2022 Counts NZ et al. *JAMA Network Open.*

eTable 2. Findings from Sensitivity Analyses: Per-Person, Discounted Results

Sensitivity Analysis	Total 5-Year Savings from PPD Prevention[a]	TCOC-based VBP (100% of 1-year savings shared)[a]	NPVoC (50% of 5-year savings shared)[a]	% Positive ROI for Payer[b]
Reduced Intervention Effectiveness	$369.16 (-73.00, 830.62)	$89.55 (-19.67, 209.64)	$184.58 (-36.50, 415.31)	76%
Reduced Costs Attributable to Depression	$360.46 (67.82, 620.77)	$88.02 (18.75, 152.69)	$180.23 (33.91, 310.38)	89%

a. An average of the difference between 1000 pairs of runs of the simulation, with each pair consisting of a run with PPD prevention implemented and one without, and the 95% credible interval. For 100% shared savings at three months and one year, our calculation reflects a distribution of potential payments from actual savings. For 50% shared savings over five years, the average payment amount reflects the suggested upfront NPVoC-based payment, and the calculation also offers the underlying distribution from which that payment amount was determined. For 100% shared savings over five years, the calculation represents the total net present value of the preventive intervention.
b. Calculated by proportion of pairs of 1000 runs for which there are still savings remaining after subtracting the upfront 50% shared savings clinician incentive amount.

© 2022 Counts NZ et al. *JAMA Network Open.*

eAppendix 4: Discussion of Sensitivity Analyses

In both sensitivity analyses, the shared savings incentives across VBP approaches were approximately half as much as in the base model. The sensitivity analysis of reduced intervention effectiveness featured greater variability in estimates than the sensitivity analysis of reduced costs attributable to depression. With no churn, both sensitivity analyses still found substantial clinician incentives for the NPVoC-based VBP approach and a high likelihood of positive return on investment (ROI) for the payer. At higher levels of churn or with incomplete intervention penetration, it is likely that much of the advantage of NPVoC-based VBP would be lost.

In current real-world settings with frequent churn, a model comparing potential NPVoC-based and TCOC-based incentives and likelihood of positive ROI would need to be parameterized with data and assumptions closer to the actual populations served and interventions implemented, in order to ensure that NPVoC-based VBP would offer meaningful returns for clinicians and high likelihood of ROI for payers. In settings with low churn (e.g. Medicaid expansion and only one or two Medicaid payers), the sensitivity analyses indicate some robustness to model mis-parameterization and the likelihood of meaningful NPVoC-based VBP returns are higher.

© 2022 Counts NZ et al. *JAMA Network Open.*

eReferences

[1] Hahn-Holbrook J, Cornwell-Hinrichs T, Anaya I. Economic and health predictors of national postpartum depression prevalence: a systematic review, meta-analysis, and meta-regression of 291 studies from 56 countries. Frontiers in psychiatry. 2018 Feb 1;8:248.
[2] Bauman BL, Ko JY, Cox S, D'Angelo DV, Warner L, Folger S, Tevendale HD, Coy KC, Harrison L, Barfield WD. Vital signs: Postpartum depressive symptoms and provider discussions about perinatal depression—United States, 2018. Morbidity and Mortality Weekly Report. 2020 May 15;69(19):575.
[3] Wisner KL, Sit DK, McShea MC, Rizzo DM, Zoretich RA, Hughes CL, Eng HF, Luther JF, Wisniewski SR, Costantino ML, Confer AL. Onset timing, thoughts of self-harm, and diagnoses in postpartum women with screen-positive depression findings. JAMA psychiatry. 2013 May 1;70(5):490-8.
[4] Underwood L, Waldie KE, D'Souza S, Peterson ER, Morton SM. A longitudinal study of pre-pregnancy and pregnancy risk factors associated with antenatal and postnatal symptoms of depression: evidence from growing up in New Zealand. Maternal and child health journal. 2017 Apr 1;21(4):915-31.
[5] Sunnqvist C, Sjöström K, Finnbogadóttir H. Depressive symptoms during pregnancy and postpartum in women and use of antidepressant treatment–a longitudinal cohort study. International journal of women's health. 2019;11:109.
[6] Venkatesh KK, Kaimal AJ, Castro VM, Perlis RH. Improving discrimination in antepartum depression screening using the Edinburgh Postnatal Depression Scale. Journal of affective disorders. 2017 May 1;214:1-7.
[7] Meijer JL, Beijers C, Van Pampus MG, Verbeek T, Stolk RP, Milgrom J, Bockting CL, Burger H. Predictive accuracy of Edinburgh Postnatal Depression Scale assessment during pregnancy for the risk of developing postpartum depressive symptoms: a prospective cohort study. BJOG: An International Journal of Obstetrics & Gynaecology. 2014 Dec;121(13):1604-10.
[8] Netsi E, Pearson RM, Murray L, Cooper P, Craske MG, Stein A. Association of persistent and severe postnatal depression with child outcomes. JAMA psychiatry. 2018 Mar 1;75(3):247-53.
[9] Martin JA, Hamilton BE, Osterman MJK, Driscoll AK. Births: Final Data for 2019. National Vital Statistics Reports; vol 70 no 2. Hyattsville, MD: National Center for Health Statistics. 2021
[10] Period Life Table, 2017 [Internet]. US Social Security Administration. Accessed 30 Mar 2021. https://www.ssa.gov/oact/STATS/table4c6.html
[11] Hoyert DL, Miniño AM. Maternal Mortality in the United States: Changes in Coding, Publication, and Data Release, 2018. National vital statistics reports: from the Centers for Disease Control and Prevention, National Center for Health Statistics, National Vital Statistics System. 2020 Jan;69(2):1-8.
[12] Singh GK. Trends and social inequalities in maternal mortality in the United States, 1969-2018. International Journal of Maternal and Child Health and AIDS. 2021;10(1):29.
[13] Palladino CL, Singh V, Campbell J, Flynn H, Gold K. Homicide and suicide during the perinatal period: findings from the National Violent Death Reporting System. Obstetrics and gynecology. 2011 Nov;118(5):1056.
[14] Wallace ME, Hoyert D, Williams C, Mendola P. Pregnancy-associated homicide and suicide in 37 US states with enhanced pregnancy surveillance. American journal of obstetrics and gynecology. 2016 Sep 1;215(3):364-e1.

© 2022 Counts NZ et al. *JAMA Network Open.*

[15] Turecki G, Brent DA. Suicide and suicidal behaviour. The Lancet. 2016 Mar 19;387(10024):1227-39.
[16] Mangla K, Hoffman MC, Trumpff C, O'Grady S, Monk C. Maternal self-harm deaths: an unrecognized and preventable outcome. American journal of obstetrics and gynecology. 2019 Oct 1;221(4):295-303.
[17] Goldman-Mellor S, Margerison CE. Maternal drug-related death and suicide are leading causes of postpartum death in California. American journal of obstetrics and gynecology. 2019 Nov 1;221(5):489-e1.
[18] Daw JR, Hatfield LA, Swartz K, Sommers BD. Women in the United States experience high rates of coverage 'churn' in months before and after childbirth. Health Affairs. 2017 Apr 1;36(4):598-606.
[19] Daw JR, Winkelman TN, Dalton VK, Kozhimannil KB, Admon LK. Medicaid Expansion Improved Perinatal Insurance Continuity For Low-Income Women: Study examines the impact of state Medicaid expansions on continuity of insurance coverage for low-income women across three time points: preconception, delivery, and postpartum. Health Affairs. 2020 Sep 1;39(9):1531-9.
[20] Johnston EM, McMorrow S, Thomas TW, Kenney GM. ACA Medicaid expansion and insurance coverage among new mothers living in poverty. Pediatrics. 2020 May 1;145(5).
[21] Haley JM, Johnston EM, Hill I, Kenney GM, Thomas TW. The Public Health Insurance Landscape for Pregnant and Postpartum Women [Internet]. Urban Institute. Jan 2021. Accessed 26 Jan 2022. https://www.urban.org/sites/default/files/publication/103561/the-public-health-insurance-landscape-for-pregnant-and-postpartum-women_1.pdf
[22] Goldman AL, Sommers BD. Among Low-Income Adults Enrolled In Medicaid, Churning Decreased After The Affordable Care Act: This study examines whether the Affordable Care Act's expansion of Medicaid eligibility had an impact on coverage disruptions–known as "churning"–among Medicaid enrollees. Health affairs. 2020 Jan 1;39(1):85-93.
[23] Ela EJ, Vizcarra E, Thaxton L, White K. Insurance Churn and Postpartum Health among Texas Women with Births Covered by Medicaid/CHIP. Women's Health Issues. 2021 Dec 13.
[24] Brantley E, Ku L. Continuous Eligibility for Medicaid Associated With Improved Child Health Outcomes. Medical Care Research and Review. 2021 Sep 16:10775587211021172.
[25] Curry SJ, Krist AH, Owens DK, Barry MJ, Caughey AB, Davidson KW, Doubeni CA, Epling JW, Grossman DC, Kemper AR, Kubik M. Interventions to prevent perinatal depression: US Preventive Services Task Force recommendation statement. Jama. 2019 Feb 12;321(6):580-7.
[26] O'Connor E, Senger CA, Henninger M, et al. Interventions to Prevent Perinatal Depression: A Systematic Evidence Review for the U.S. Preventive Services Task Force [Internet]. Rockville (MD): Agency for Healthcare Research and Quality (US); 2019 Feb. (Evidence Synthesis, No. 172.) Available from: https://www.ncbi.nlm.nih.gov/books/NBK537819/
[27] Washington State Institute for Public Policy. Benefit-Cost Technical Documentation [Internet]. Washington State Institute for Public Policy. Dec 2019. Accessed Sep 21, 2020. https://www.wsipp.wa.gov/TechnicalDocumentation/WsippBenefitCostTechnicalDocumentation.pdf
[28] Simpson J, Albani V, Bell Z, Bambra C, Brown H. Effects of social security policy reforms on mental health and inequalities: A systematic review of observational studies in high-income countries. Social Science & Medicine. 2021 Jan 26:113717.
[29] Boyd-Swan C, Herbst CM, Ifcher J, Zarghamee H. The earned income tax credit, mental health, and happiness. Journal of Economic Behavior & Organization. 2016 Jun 1;126:18-38.

© 2022 Counts NZ et al. *JAMA Network Open.*

[30] US Government Accountability Office. GAO-17-558: Federal low-income programs – Eligibility and benefits differ for selected programs due to complex and varied rules. June 2017. https://www.gao.gov/assets/690/685551.pdf
[31] Margerison CE, MacCallum CL, Chen J, Zamani-Hank Y, Kaestner R. Impacts of Medicaid expansion on health among women of reproductive age. American journal of preventive medicine. 2020 Jan 1;58(1):1-1.
[32] MACStats: Medicaid and CHIP Data Book. Washington, DC: Medicaid and CHIP Payment and Access Commission. Dec 2020. https://www.macpac.gov/wp-content/uploads/2020/12/MACStats-Medicaid-and-CHIP-Data-Book-December-2020.pdf
[33] Blattman C, Jamison JC, Sheridan M. Reducing crime and violence: Experimental evidence from cognitive behavioral therapy in Liberia. American Economic Review. 2017 Apr;107(4):1165-206.
[34] Postpartum Depression Screening [Internet]. American College of Obstetricians and Gynecologists. Accessed 26 Feb 2022. https://www.acog.org/practice-management/coding/coding-library/coding-question-postpartum-depression-screening
[35] Medicaid Physician Fee Index [Internet]. Kaiser Family Foundation. Accessed 26 Feb 2022. https://www.kff.org/medicaid/state-indicator/medicaid-fee-index/?currentTimeframe=0&sortModel=%7B%22colId%22:%22Location%22,%22sort%22:%22asc%22%7D
[36] Procedure Fee Files & CPT Codes [Internet]. Virginia Medicaid. Accessed 26 Feb 2022. https://www.dmas.virginia.gov/for-providers/procedure-fee-files-cpt-codes/
[37] Dagher RK, McGovern PM, Dowd BE, Gjerdingen DK. Postpartum depression and health services expenditures among employed women. Journal of occupational and environmental medicine. 2012 Feb 1;54(2):210-5.
[38] Greenberg PE, Fournier AA, Sisitsky T, Pike CT, Kessler RC. The economic burden of adults with major depressive disorder in the United States (2005 and 2010). The Journal of clinical psychiatry. 2015 Feb 25;76(2):155-62.
[39] Witt WP, Weiss AJ, Elixhauser A. Overview of hospital stays for children in the United States, 2012. Heathcare Cost and Utilization Project (H-CUP). Statistical Brief #187. December 2014. Available at: https://www.hcup-us.ahrq.gov/reports/statbriefs/sb187-Hospital-Stays-Children-2012.jsp
[40] Farr SL, Dietz PM, Rizzo JH, Vesco KK, Callaghan WM, Bruce FC, Bulkley JE, Hornbrook MC, Berg CJ. Health care utilisation in the first year of life among infants of mothers with perinatal depression or anxiety. Paediatric and perinatal epidemiology. 2013 Jan;27(1):81-8.
[41] Epperson CN, Huang MY, Cook K, Gupta D, Chawla A, Greenberg PE, Eldar-Lissai A. Healthcare resource utilization and costs associated with postpartum depression among commercially insured households. Current Medical Research and Opinion. 2020 Oct 2;36(10):1707-16.
[42] Khan HA, Knusel KD, Calabrese JR, Salas-Vega S. Direct incremental healthcare costs associated with mood disorders in the United States, 2007–2017. Journal of Affective Disorders. 2020 Aug 1;273:304-9.
[43] Pilon D, Sheehan JJ, Szukis H, Singer D, Jacques P, Lejeune D, Lefebvre P, Greenberg PE. Medicaid spending burden among beneficiaries with treatment-resistant depression. Journal of comparative effectiveness research. 2019 Apr;8(6):381-92.
[44] Incerti D, Jansen JP. hesim: Health Economic Simulation Modeling and Decision Analysis. arXiv preprint arXiv:2102.09437. 2021 Feb 18.

© 2022 Counts NZ et al. *JAMA Network Open.*

[45] Naimark DM, Kabboul NN, Krahn MD. The half-cycle correction revisited: redemption of a kludge. Medical Decision Making. 2013 Oct;33(7):961-70.

© 2022 Counts NZ et al. *JAMA Network Open.*
